# Supplementary material for: Balloon-Expandable versus Self-Expandable Valves in Transcatheter Aortic Valve Implantation: Complications and Outcomes from a Large International Patient Cohort
Source: J Clin Med. 2021 Sep 4;10(17):4005. doi: 10.3390/jcm10174005 (PMC8432462; doi:10.3390/jcm10174005)
Supplement: Supplementary file 1 [file jcm-10-04005-s001.zip › jcm-1318319-supplementary.pdf]

Supplementary material

Supplementary table S1

Supplementary table S2

Supplementary table S3

Supplementary Table S1. Overview of original studies, number of patients, proportion of balloon- and self-expandable valves, procedure of valve selection and aortic valve calcification as measured by computed tomography.

Study name and PMID	CENTER* (n=)	BE vs SE* (%)	Valve selection	Calcification on CT
**Brazilian TAVI registry** 27496637	768	BE: 195 (25%) SE: 573 (75%)	Heart team in different centers	Not available
**FRANCE-2** 25240554	2347	BE: 1506 (64%) SE: 841 (36%)	Heart team in different centers	8% porcelain aorta
**Milano** 27184169	515	BE: 270 (52%) SE: 245 (48%)	Multidisciplinary heart team	AV calcification: 52.6% grade 1-2, 46.6% grade 3-4. Mean annulus diameter 23.0 mm.
**Verona** 27621826	346	BE: 254 (73%) SE: 92 (27%)	Institutional Heart Team	Not available
**Rabin** 27726854	544	BE: 120 (22%) SE: 424 (78%)	Institutional Heart Team	Not available
**Padova** 26603025	447	BE: 352 (79%) SE: 95 (21%)	Institutional Heart Team	16.3% porcelain aorta
**Spanish TAVI registry** 24774108	5320	BE: 2451 (46%) SE: 2869 (54%)	Heart team in different centers	Mean annulus diameter 23.2 mm
**BRAVO-3** 26477635	732	BE: 500 (68%) SE: 232 (32%)	Institutional Heart Team	Not available
**WIN-TAVI** 27491609	785	BE: 366 (47%) SE: 419 (53%)	Institutional Heart Team	AV calcification: 17.7% grade 1-2, 82.3% grade 3-4. Mean annulus diameter 21.6 mm.
**OBSERVANT** 26271063	577	BE: 225 (39%) BE: 352 (61%)	Heart team in different centers	Patients with porcelain aorta were excluded. Mean annulus diameter 22.2 mm.

** number of patients from the original study included in the CENTER database. CT = computed tomography.*

Supplementary table S2. Baseline characteristics of the overall patient population and the propensity matched cohort

	Overall patient population			Propensity matched population		
	BE(n=6239)	SE(n=6142)	SMD	BE(n=4096)	SE(n=4096)	SMD
**Demographics**						
Age (years)	81.7±6.9	81.2±7.1	0.071	81.5±7.1	81.3±7.1	0.028
Female gender	3700(59)	3420(56)	0.081	2364(58)	2336(57)	0.015
Body mass index (kg/m^2)	27.0±4.8	27.3±4.9	0.062	27.2±4.8	27.1±4.8	0.021
**Medical history**						
Previous CVA or TIA	660(11)	631(10)	0.018	420(10)	436(11)	0.023
Previous MI	815(13)	855(14)	0.041	558(14)	574(14)	0.018
Previous PCI	1239(20)	1421(23)	0.107	883(22)	886(22)	0.002
Previous CABG	728(12)	745(12)	0.024	497(12)	511(13)	0.018
Diabetes mellitus	1952(31)	1924(31)	0.043	1298(32)	1293(32)	0.003
Hypertension	4920(79)	4815(78)	0.015	3223(79)	3231(79)	0.006
Dyslipidemia	3383(54)	3410(56)	0.029	2236(55)	2247(55)	0.007
Peripheral artery disease	992(16)	816(13)	0.116	588(14)	599(15)	0.012
Coronary artery disease	2552(41)	2530(41)	0.007	1668(41)	1682(41)	0.009
Atrial fibrillation	1764(28)	1590(26)	0.071	1087(27)	1115(27)	0.019
GFR<30 ml/min	855(14)	827(14)	0.011	565(14)	574(14)	0.011
Logistic euroSCORE(%)	15.2(10.0-23.1)	14.6(9.0-22.7)	0.055	15.0(9.7-23.0)	15.0(9.3-23.3)	0.018
STS-PROM (%)	6.4(4.0-13.9)	6.4(3.9-12.3)	0.082	6.3(4.0-14.4)	6.6(4.0-12.8)	0.051
Aortic mean gradient	51.1±17.5	51.0±17.2	0.006	51.0±17.6	51.1±17.4	0.006
**Implanted valves**						
Third generation	1713(30)	1670(29)	0.059	1122(30)	1091(28)	0.042

*A standardised mean difference of 0.1 or less indicated a negligible difference between the means of the two cohorts. *in mmHg.*
CVA= cerebrovascular event. TIA=transient ischemic attack. MI=myocardial infarction. PCI= percutaneous coronary intervention. CABG= coronary artery bypass graft.
GFR=Glomerular Filtration Ratio. STS-PROM=Society of Thoracic Surgeons Predicted Risk of Mortality. EuroSCORE= European System for Cardiac Operative Risk Evaluation.

Supplementary Table S3. Baseline and procedural characteristics of patients that completed one year follow-up compared to those that did not complete one year follow-up in the propensity matched population

	Completed one year follow-up (n=5410)	Did not complete one-year follow-up (n-2782)	*p-value*
**Demographics**			
Age (years)	81.3±7.0	81.6±7.3	0.07
Female gender	3239(60)	1461(53)	<0.001
Body mass index (kg/m^2)	27.1±4.9	27.2±4.6	0.28
**Medical history**			
Previous CVA or TIA	569(11)	287(10)	0.78
Previous MI	740(14)	392(14)	0.61
Previous PCI	1179(22)	590(21)	0.54
Previous CABG	668(12)	340(12)	0.87
Diabetes mellitus	1736(32)	855(31)	0.21
Hypertension	4325(80)	2129(77)	<0.001
Dyslipidemia	3046(56)	1437(52)	<0.001
Peripheral artery disease	795(15)	392(14)	0.46
Coronary artery disease	2184(40)	166(42)	0.18
Atrial fibrillation	1467(27)	735(26)	0.50
GFR<30 ml/min	809(15)	330(12)	<0.001
Logistic euroSCORE(%)	14.6(9.2-23.0)	15.7(10.0-23.3)	0.008
STS-PROM (%)	6.2(4.0-12.4)	7.0(4.1-15.5)	<0.001
Aortic mean gradient	50.o±16.9	53.1±18.4	<0.001
Aortic max gradient	79.6±23.8	78.7±22.6	0.26
Aortic valve area (cm^2)	0.65±0.20	0.67±0.19	0.002
**Implanted valves**			
Balloon-expandable	2360(44)	1736(62)	<0.001
Third generation	1405(26)	808(35)	<0.001

CVA= cerebrovascular event. TIA=transient ischemic attack. MI=myocardial infarction. PCI= percutaneous coronary intervention. CABG= coronary artery bypass graft. GFR=Glomerular Filtration Ratio. STS-PROM=Society of Thoracic Surgeons Predicted Risk of Mortality. EuroSCORE= European System for Cardiac Operative Risk Evaluation.
